# Steroid-Induced Tumor Lysis Syndrome Accompanied by Diabetic Ketoacidosis and Acute Renal Failure in a Non-Hodgkin Lymphoma Patient

**DOI:** 10.7759/cureus.24491

**Published:** 2022-04-26

**Authors:** Kevin Cao, Jesse C Wu, Michelle Hernandez, Latha Ganti

**Affiliations:** 1 Internal Medicine, Einstein Medical Center Montgomery, East Norriton, USA; 2 Emergency Medicine, University of Central Florida College of Medicine, Orlando, USA; 3 Emergency Medicine, Hospital Corporation of America (HCA) Florida Osceola Hospital, Kissimmee, USA; 4 Emergency Medicine, Envision Physician Services, Plantation, USA

**Keywords:** hypochloremia, hypercalcemia, emergency department, non-hodgkins lymphoma, tumor-lysis syndrome

## Abstract

We present a case of a 66-year-old male with a past history of newly diagnosed non-Hodgkin lymphoma, diabetes, and recent surgical splenectomy secondary to splenic infarct who presented to the Emergency Department (ED) with several nonspecific symptoms that were consistent with tumor lysis syndrome. This case report discusses the clinical presentation, diagnosis, and management of spontaneous tumor lysis syndrome.

## Introduction

Tumor lysis syndrome (TLS) is a rare emergent syndrome that results in the release of intracellular contents from cell lysis that occurs quickly. TLS is often triggered by chemotherapy but may also occur due to radiation, steroids, or spontaneously. Typically, tumor lysis occurs in patients with tumors with a significant proliferation rate, tumor burden, or sensitivity to cytotoxic therapy. TLS is commonly seen in those with hematological neoplasms such as acute lymphoblastic leukemia (ALL) and acute myeloid leukemia (AML). However, lysis may also happen in those with high-grade lymphomas such as Burkitt’s Lymphomas and other solid tumors [1,2]. Intermediate grade non-Hodgkin lymphoma (NHL), such as diffuse large B cell lymphoma, can also be at high or intermediate risk for tumor lysis. Comparatively, slower dividing low-grade lymphoma or Hodgkin’s lymphoma are at lower risk of tumor lysis. Currently, there are only sparse case reports of TLS in low-grade and intermediate-grade NHL, Hodgkin's disease, AML and ALL in blast crisis, and myeloproliferative disorders [3,4].

Spontaneous tumor lysis syndrome (STLS) is an oncologic emergency initiated by releasing copious amounts of potassium, phosphate, nucleic acids, and uric acid into systemic circulation by extensive tumor breakdown. Lysis in STLS occurs without any antineoplastic agents or interventions such as chemotherapies [5]. As the tumors are catabolized, the tumor cells release electrolytes and nucleic acids into circulation. The nucleic acids are further broken down into uric acids, resulting in hyperuricemia. Uric acid can collect in renal tubules and cause an intrarenal obstruction due to the acidity [2]. The excessive level of these products may result in severe consequences such as acute renal injury, arrhythmias, altered mental status, seizures, and potentially death [1]. 

TLS can also be triggered by steroid treatment. There are a few cases in the literature documenting steroid-induced tumor lysis syndrome in lymphoma, causing acute renal failure [6,7] and metabolic acidosis [8,9]. However, cases that report steroid-induced TLS from lymphoma causing diabetic ketoacidosis (DKA) and acute renal failure are lacking.

Early recognition of metabolic derangements, rapid treatment, and early coordination of care with the oncology, nephrology, and intensive care team is critical for the care of a potentially lethal syndrome. Even in those with less prone to malignancies, including TLS as a differential diagnosis is imperative. Therefore, it is crucial for the emergency medicine physician to be aware of this life-threatening metabolic emergency and its associated complications and how to manage them.

## Case presentation

The patient is a 66-year-old male who presents to the ED for shortness of breath, nausea, generalized abdominal pain, bone pain, decreased appetite, oral intake, and decreased urine output for the past three days. He has a past medical history of diabetes, non-Hodgkin lymphoma that was newly diagnosed four weeks before presentation, and splenectomy secondary to splenic infarct of unclear etiology three months prior to presentation. A month prior, the patient was seen for cervical lymphadenopathy and was subsequently diagnosed with non-Hodgkin lymphoma after cervical lymph node biopsy. The patient reported subjective fever and unintentional weight loss of approximately 30 pounds over the past three months. He denies coughing, vomiting, chest pain, or diarrhea. He endorses insomnia due to the symptoms for the past three days. During the interview with the patient, he stated that he had not started his chemotherapy for the lymphoma. He was instructed to take prednisone for 10 days but stop his metformin and insulin PM three days ago for mediport insertion today. However, this procedure was canceled due to his symptoms. On exam, the patient is ill-appearing with dry mucous membrane and epigastric and mid-abdominal tenderness. The patient was tachycardic, but otherwise, all other vital signs were stable. He was afebrile.

On initial workup, laboratory studies revealed that the patient was mildly hyponatremic, hyperkalemic, hypochloremic, with an elevated anion gap, elevated blood urea nitrogen (BUN) and creatinine, hypercalcemia, hyperphosphatemia, elevated aspartate aminotransferase (AST) and hypoalbuminemia, elevated beta-hydroxybutyrate, and hyperglycemia (Table 1).

**Table 1:** Laboratory values pH: potential of hydrogen. pCO2: partial pressure of carbon dioxide. pO2: partial pressure of oxygen. BHB: beta-hydroxybutyrate. HCO3: bicarbonate. O2 Sat: oxygen saturation

Arterial blood gas (left radial artery)				
pH		7.35 - 7.45		7.44
pCO2		35 - 48 mmHg		19 L
pO2		83 - 108 mmHg		87.1
HCO3		21 - 28 mmol/L		13.1 L
O2 Sat		94 - 98 %		97.3
Base Excess		-2 - 3 mmol/L		-8.9 L
Allen Test				Positive
Chemistry				
Sodium		(136 - 145 mmol/L)		128 L
Potassium		(3.7 - 5.1 mmol/l)		6.9 C
Chloride		(98 - 107 mmol/L)		93 L
Carbon Dioxide		(21 - 32 mmol/l)		11 L
Anion Gap				30.9
Blood Urea Nitrogen		(7 - 18 mg/dl)		72 H
Creatinine		(0.55 – 1.3 mg/dl)		2.34 H
Glucose		74 - 106 mg/dl		282 H
Calcium		8.4 - 10.1 mg/dl		8.5
Phosphorus		2.5 - 4.9 mg/dL		7.6 H
Total Bilirubin		0.2 - 1.5 mg/dl		0.9
Aspartate Aminotransferase		10 - 37 unit/L		99 H
Alanine Aminotransferase		12 - 78 unit/L		26
Alkaline Phosphatase		45 - 117 unit/L		139 H
Creatinine Kinase		35 - 232 unit/L		111
Troponin I		<0.05 ng/mL		0.05 H
Total Protein		6.4 - 8.2 g/dL		6.5
Albumin		3.4 - 5.0 g/dl		2.1 L
BHB		0.2 - 2.8 mg/dL		21.4 H
Glucose		74 - 106 mg/dL		291 H
Lactic Acid		0.4 - 2.0 mmol/L		9.7 H
Uric Acid		3.5 - 7.2 mg/dL		11.5H
Lactate Dehydrogenase		100 - 190 unit/L		3011 H
Hematology				
White Blood Cell Count	4.0 - 10.5 10^3/u		58.0 H	
Red Blood Cell Count	4.63 – 6.00 10^6/uL		6.05 H	
Hemoglobin	13.7 - 17.5 g/dL		14.9	
Hematocrit	34.1 – 44.9 %		45.5 H	
Mean Corpuscular Volume	79.4 - 94.8 fL		75.2 L	
Mean Corpuscular Hemoglobin	25.6 - 32.2 pg		24.6 L	
Mean Corpuscular Hemoglobin Concentration	32.2 - 35.5 g/dL		32.7	
Red Cell Distribution Width	11.6 - 14.4 %		23.6 H	
Platelet Count	150 - 450 10^3/uL		77 L	
Segmented Neutrophils	38.0 - 74.0 %		34 L	
Band Neutrophils	5.0 - 11.0 %		8	
Lymphocytes	20 - 45 %		9 L	
Monocytes	3 - 10 %		24 H	
Eosinophils	0 - 4 %		0	
Basophils	0 - 2.0 %		0	
Metamyelocytes	0 - 2 %		3 H	
Myelocytes	0 - 0 %		11 H	
Promyelocytes	0 - 0 %		1 H	
Nucleated Red Blood Cells	0 - 0 %		6 H	
Atypical Lymphocytes	0 - 1 %		2 H	
Platelet Estimate	Adequate		Decreased	
Platelet Morphology	Normal		Occasional giant platelets	
Morphology Comment	Red blood cells appear normal			

Complete blood count demonstrated leukocytosis and thrombocytopenia. Blood peripheral smear revealed immature granulocytes consisting of metamyelocytes, promyelocytes, and myelocytes. Additional laboratory studies also showed lactic Acidosis, hyperuricemia, and elevated lactate dehydrogenase. Arterial blood gas revealed a pH of 7.44, pCO2 of 19, pO2 of 87, and bicarbonate of 13. His COVID-19 test was negative. Presumptive diagnoses at this point were diabetic ketoacidosis due to steroids, tumor lysis syndrome complicated by acute renal failure and hyperkalemia, and sepsis. The patient was started on intravenous fluids for acute renal failure. The patient was then started on calcium gluconate, insulin infusion, and D5-NS to treat hyperkalemia and Acidosis. Ceftriaxone was initiated for presumptive sepsis. The patient was then admitted to the intensive care unit (ICU) for further management and oncology consultation, as his presentation was suggestive of STLS.

During hospital day one, the patient developed worsening hypocalcemia and Acidosis. It was determined that he would need to be stabilized prior to initiating mediport insertion and treatment of his non-Hodgkin lymphoma. On hospital day two, the patient became anuric, confused, and lethargic. Laboratory studies revealed improved hyperkalemia, a persistently high anion gap, and Acidosis despite insulin drip and IV fluid. However, he then began to develop pulmonary edema. Nephrology was consulted, and the patient was started on hemodialysis due to worsening renal function and volume overload. Rasburicase was also started to manage the elevated uric acid level. On hospital day three, the patient had persistent worsening renal function, anuria, metabolic Acidosis, and volume overload despite hemodialysis. He later became hypotensive, requiring vasopressor support in the ICU. The patient had newly developed atrial fibrillation with rapid ventricular response. On hospital day four, due to his poor prognosis, the patient was made "do-not-resuscitate" and discharged to hospice for comfort care.

## Discussion

TLS typically occurs after treatment of malignancy but may occur commonly after steroid treatment or spontaneously in rare cases prior to treatment. Non-Hodgkin lymphomas are a heterogeneous group of solid tumors. Caveats for lysis for solid tumors include bulky solid tumors or high sensitivity to cytotoxic therapies. Through a literature review, STLS in solid tumors is seen in a few cases, as demonstrated by its low likelihood for this syndrome [10]. Most cases of lysis seen in solid tumors were due to the initiation of radiation, chemotherapy, or steroids. Our patient had undergone a 10-day course of steroids, which is most likely the potential culprit as he has not started his chemotherapy. Current guidelines and literature reveal that most solid tumors are at low risk for spontaneous lysis. The occurrence of both TLS and STLS is typically rare for those with NHL, but certain features can make NHL at high risk for TLS. Certain tumor types and burden make NHL particularly high risk for TLS, such as advanced Burkitt’s lymphoma, early Burkitt’s lymphoma with high LDH, and diffuse large B-cell lymphoma with high LDH [11]. Pre-existing conditions such as renal dysfunction can also make an NHL patient at high risk for TLS. We do not know this patient’s NHL subtype, its tumor stage, disease burden, or baseline LDH, so it is difficult to risk stratifying this patient for TLS based on these tumor-specific features. However, we do know that this patient is at high risk for renal dysfunction given his diabetes history despite normal baseline creatinine. His steroid use likely exacerbated his hyperglycemia, subsequently leading to DKA and triggering TLS at the same time. The combination of these two conditions likely worsened his renal function, resulting in his renal failure and ultimate poor outcome.

STLS requires clinical correlation with the patient's known malignancy, the likelihood for lysis, and laboratory studies. Current guidelines reflect that of the Cario-Bishop guidelines [12,13]. If the laboratory studies reveal at least two abnormalities, the patient may be diagnosed with TLS. Abnormalities noted by current Cario-Bishop guidelines classify patients with TLS or STLS have a serum uric acid level ≥8 mg/dL, or a 25% increase from baseline; a serum potassium level ≥6 mmol/L, or a 25% increase from baseline; serum phosphate levels ≥4.5 mg/dL in adults, or a 25% increase from baseline; and a serum calcium level ≤7 mg/dL, or a 25% decrease from baseline [12-14] In addition, these abnormalities must have occurred three days prior to or within seven days following the beginning of chemotherapy treatment to be considered for a diagnosis of TLS [12,13]. However, as this patient has not started any cytotoxic therapy, the evidence is clear that the patient was experiencing steroid-induced TLS given recent steroid use.

Aggressive IV fluid administration is the key to preventing TLS. The aim is to optimize renal perfusion with a target urine output of 2 ml/kg [15]. Adequate glomerular filtration and urinary output (UOP) are important in preventing precipitations of uric acid crystals or calcium phosphate deposits in the renal tubules, one of the main mechanisms behind acute renal failure in TLS patients. However, in vitro fertilization (IVF) administration also puts patients at risk for volume overload in the setting of preexisting acute kidney injury. In this case, reversing the underlying cause of acute kidney injury (AKI) should also be addressed. Our patient presented with TLS and AKI that gradually progressed to acute renal failure. IVF administration ultimately contributed to pulmonary edema in this patient due to poor renal function. Dialysis then had to be initiated due to volume overload, persistent electrolytes abnormalities, and acidosis. Diuretics such as furosemide could be a feasible therapeutic option for volume overload, but it is unlikely to work in patients that are already in renal failure and are anuric.

Uric acid-lowering agents, such as allopurinol (xanthine oxidase inhibitor) and rasburicase (urate oxidase), are additional key management strategies for preventing TLS. Allopurinol prevents the new uric acid formation and thus has the detrimental potential for obstructive AKI. However, allopurinol does not reduce pre-existing high uric acid levels, as seen in our patient. Therefore, it was not the best option in this case. In addition, allopurinol can also precipitate xanthine crystals in the renal tubules, worsening renal function. In patients at high risk for TLS with pre-existing high uric acid levels and those with impaired renal function, rasburicase is recommended. Rasburicase lowers the serum uric acid level by converting it to a more water-soluble compound, allantoin [15]. Two prospective trials have demonstrated the efficacy of rasburicase at reducing serum uric acid levels in patients at high risk for TLS [16-17]. Rasburicase should be started within the first 24 hours if possible [10,18]. In our patient, his TLS was further complicated by acute renal failure and DKA, making clinical management much more difficult. His clinical deterioration was progressing too quickly for rasburicase to have any meaningful clinical effect.

ED management includes IV hydration and stabilization of metabolic derangements. As the patient will have a variety of laboratory abnormalities, correction of elevated potassium, creatinine, and uric acid levels take precedence. Hyperkalemia is known to cause arrhythmias, as seen by the patient developing new-onset atrial fibrillation during his hospitalization. Uric acid levels further worsen the patient's acute renal failure. Acidosis may also be present. Nephrology consultation for hemodialysis may be required if renal function and acidosis do not improve. Oncology should also be consulted for further guidance, although limitations from TLS will further hold off treating the malignancy until the patient is stabilized.

Despite limited findings in the literature, research reveals that the prognosis of STLS and TLS is typically viewed as poor and remains guarded. Current research findings reveal that AKI is the strongest predictor of short-term and long-term mortality despite potentially confounding variables such as the malignancy itself and other pre-existing comorbidities [19]. Pre-existing conditions such as diabetes pose an additional layer of complexity in managing STLS and TLS. Cancer patients with diabetes tend to have higher mortality [20]. Patients with any malignancy and who are experiencing DKA will have poorer survival chances due to the sequela from the electrolyte imbalances during their hospitalization.

## Conclusions

TLS is a condition that requires clinical acumen and immediate action. Although STLS is rare in those with non-Hodgkin lymphoma, this syndrome should still be considered as a differential for an acute oncologic emergency. Clinical correlation of laboratory studies with a thorough history is essential in elucidating the patient's status and overall disposition. IV fluid administration, correcting electrolytes abnormalities, and lowering serum uric acid levels should be the cornerstone of TLS therapy. Dialysis may be required if electrolyte abnormalities, acidosis, volume overload, and uremia do not resolve with medical management. Oncology, nephrology, and intensive care specialists should be engaged early to assist with the management of TLS patients.
